# Designing of metallic nanocrystals embedded in non-stoichiometric perovskite nanomaterial and its surface-electronic characteristics

**DOI:** 10.1038/s41598-017-09031-5

**Published:** 2017-08-21

**Authors:** Jagadeesh Suriyaprakash, Y. B. Xu, Y. L. Zhu, L. X. Yang, Y. L. Tang, Y. J. Wang, S. Li, X. L. Ma

**Affiliations:** 1 Shenyang National Laboratory for Materials Science, Institute of Metal Research, Chinese Academy of Sciences, 72 Wenhua Road, 110016 Shenyang, China; 2 University of Chinese Academy of Sciences, 100039 Beijing, China; 3 State Key Laboratory of Advanced Processing and Recycling of Non-ferrous Metals, Lanzhou University of Technology, 730050 Lanzhou, China

## Abstract

Engineering of novel functional nanocomposite as like as the metallic nanocrystals supported non-stoichiometric perovskite nanomaterial in controlled parameters (size, shape and ratio of chemical characteristics) is a challengeable task. In this context, we present a facile route to fabricate and study its physicochemical property at real time mode in this report. Nanoscale pure Pb crystals surfaced on non-stoichiometric A-site deficient Pb_1-x_TiO_3-y_ nanoparticle were fabricated when a precursor lead titanate (PbTiO_3_) nanoparticle was exposed to an electron beam irradiation (EBI) in a transmission electron microscope (TEM) at ambient temperature. In the state of the art, the chemical states and electronic structure of non-irradiated and irradiated PbTiO_3_ were studied by X-ray photoelectron spectroscopy (XPS). Electron bombardment resulted in a new visible feature at low binding energy in the Pb 4f core level, while Ti 2p and O 1s line shape showed slight changes. The Fermi level of the corresponding materials was determined to be 1.65 ± 0.1 eV and 2.05 ± 0.1 eV above the valence band maximum, respectively. The normal, weakly p-type PTO exhibits peculiar n-type feature after EBI process (The Fermi level moves near to the conduction band). A feasible mechanism is proposed involving the electron-stimulated local bond-breaking phenomenon in PbTiO_3_.

## Introduction

Perovskite type ABO_3_ materials are of immense interest due to their potential application, especially titanates ATiO_3_ (Where A = Ba, Sr, Ca, Pb) family exhibits extraordinary optical, electrical and magnetic properties in both bulk and nanoscale region^1–6^. Considerable research has been focused on the fabrication of these multifunctional materials from the last three decades achieved by modification of the materials (structure, shape, composition, defects and etc)^7–10^. Growing interest has been propelled in the probe of non-stoichiometric transition metal titanates (xA-A_1-x_TiO_3-y_) materials. Defected and impurities implanted (doping) perovskites have been studied intensively. A_1-x_B_x_TiO_3-y_ (Where A and B are transition or alkali metals; A≠B) type materials are synthesised by physical as well as chemical methods in earlier studies^11–26^. Despite the fact that perovskite nanomaterials have been extensively investigated over the past decade, to the best of our knowledge, the fabrication of A-site void xA-A_1-x_TiO_3-y_ type nanocomposite by electron beam irradiation, surface characteristics and its formation mechanism has not been described thus far. The new nanoscale structures interacting with electron beam has been primarily studied by transmission electron microscopy (TEM). *In situ* observation study of electron and matter interaction has been carried out in metals (Au, Pt, In, Ag, Mo, Bi, Ni and Li)^27–32^, metal oxides (Li_2_O, WO_3_, V_2_O_5_, Fe_3_O_4_)^33–35^, semiconductors (Si, Ge, GaP, GaAs, CdS, InN, ZnS)^36–40^, carbon nanotube (CNT)^41, 42^ and ceramics (Pb-PST, BaTiO_3_, LaPO_4_, ScPO_4_)^43–47^. The main purpose of this work is to address the question of the role of electron beam irradiation on the ABO_3_ nanomaterial and its electronic structure modification. In addition, to reveal the formation mechanism of the novel metal supported non-stoichiometric xA-A_1-x_TiO_3-y_ type nanomaterials is also important. We chose PbTiO_3_ (PTO) as a prototypical material because its lattice parameters, phase transition, bonding nature and mass value are simple. Besides, PTO is a classic example of ferroelectric perovskite with high tetragonality and it exhibits a unique negative thermal expansion in the perovskite family, which has been widely used both in academic research and device application. The large ionic shift leads to a large spontaneous polarization, which is in fact among the largest in the perovskite family. Moreover, a double layer perovskite system, Bi_4_Ti_3_O_12_ (BiTO) also utilized for sake of the reliability of this innovative technique.

In this article, we report a simple method to fabricate the pure Pb nanoparticles supported on non-stoichiometric PbTiO_3_ nanomaterial by electron beam irradiation. The growth mechanism of the metal nanoparticles and its structural stability were investigated. An x-ray photoelectron spectroscopy study revealed the surface characteristic as well as electronic structure of the non-irradiated and irradiated PTO nanomaterials. This study disclosed that the electron irradiation can be utilized as one step route to fabricate the novel xA-A_1-x_TiO_3-y_ perovskite nanocomposite and to get the knowledge of dynamic behaviour of an unstable nanoscale perovskite material in TEM, which leads to understanding of the dynamics of phase transformations and the atomistic nature of bonding. In addition, we observed the same phenomenon in the BiTO material (See Supplementary Information).

## Results

### Morphology and crystal structure of PbTiO_3_ and xPb-Pb_1-x_TiO_3-y_ nanostructures

**Figure 1:**
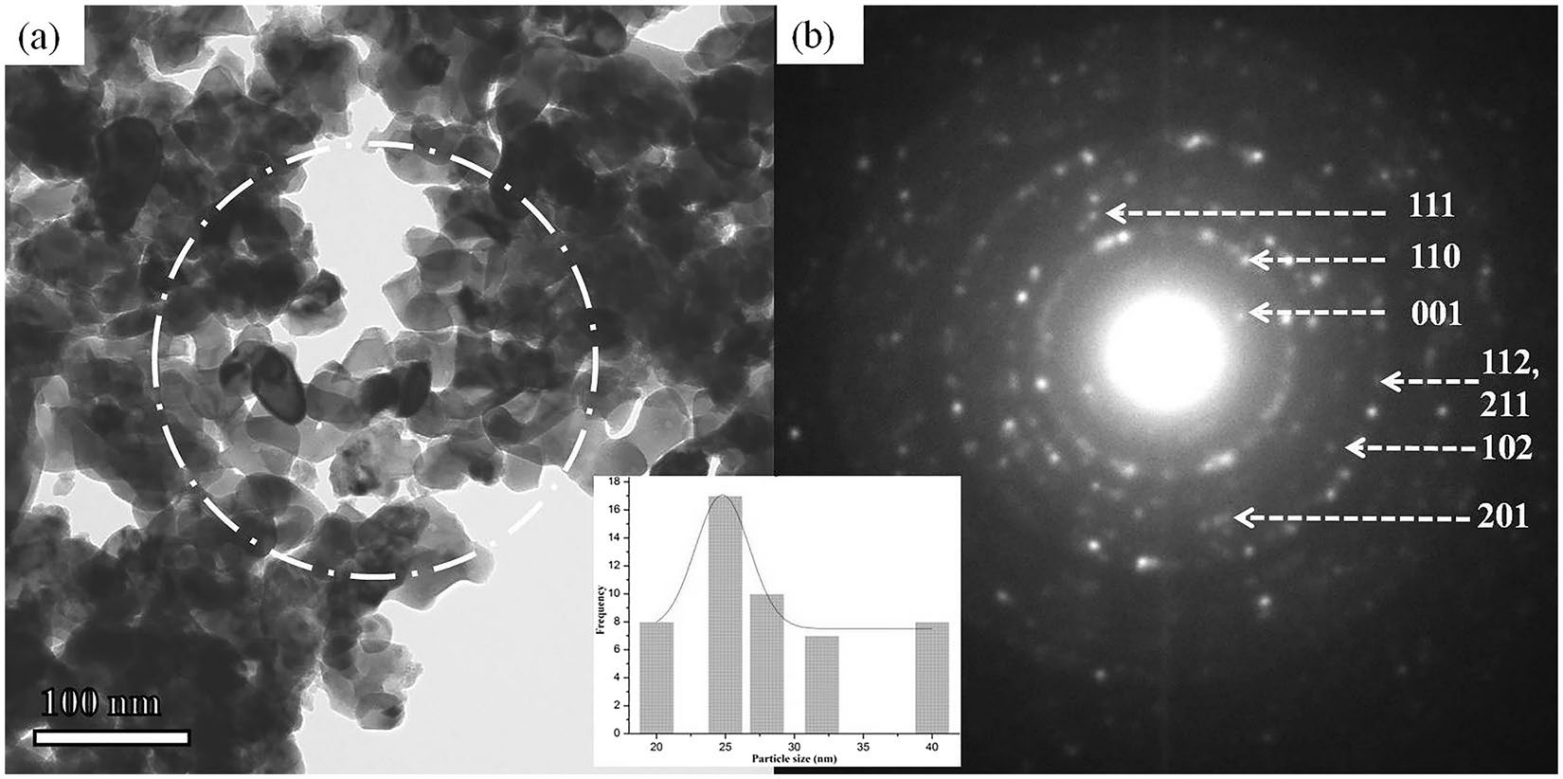
(**a**) Low magnification TEM image of as-prepared PTO sample, (**b**) Corresponding SAED pattern of circled area. The inset is a size distribution curve of the particles.

To provide more insight into the structural and the morphology of the synthesised material, we carried out XRD as well as TEM studies before the irradiation process. Figure 1(a) shows the TEM images of the as-synthesised sample. The shapes of the most particles are spherical with the particle size of 18-40 nm. Few particles are found to be clustered in a chain due to agglomeration of the particles. The selected area electron diffraction pattern (SAED) taken over a large area indicative in a white circle in Fig. 1(a) illustrated that polycrystalline PbTiO_3_ is present. The SAED pattern of Fig. 1(b) reveals diffraction rings indicative of the random crystallographic orientations of the nanocrystal of the synthesised material. All diffraction rings are indexed as those of a tetragonal structure of PTO consistent with the XRD data (See Supplementary Information Fig. S1).

**Figure 2:**
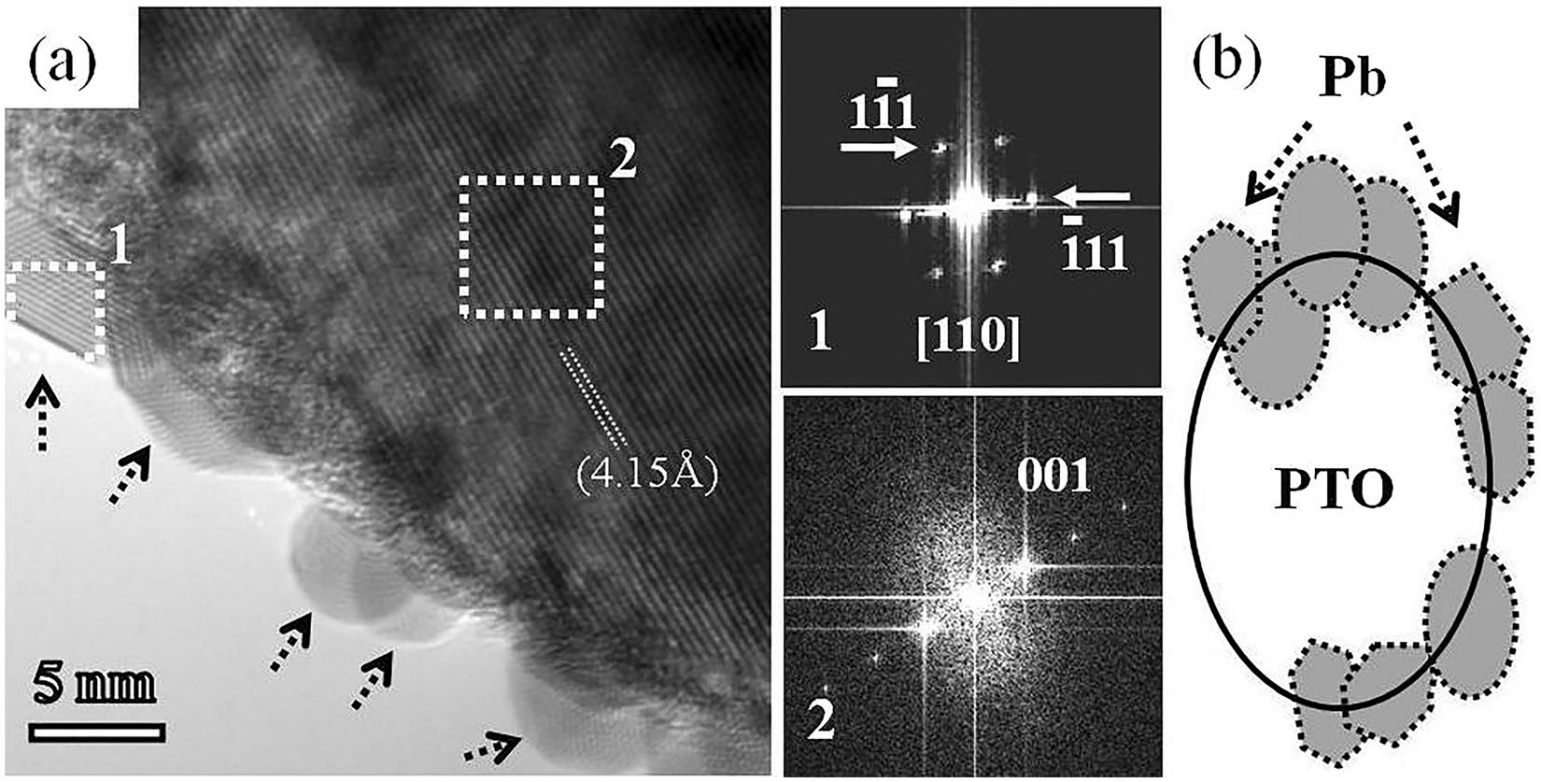
(**a**) HRTEM image of Pb-NPTO nanomaterial. The right side image 1 and 2 are corresponding FFT pattern of Pb and PTO, respectively (Where the selected area shown in (**a**) by white dotted rectangles). (**b**) A model diagram portrays the Pb-PTO coexisting structure.

We found that the pure Pb nanoparticles supported on NPTO (here after NPTO refers non-stoichiometric PbTiO_3_) nanoparticle could be synthesised by electron beam irradiation of PbTiO_3_ nanoparticle. Intriguingly, The Pb metal nanoparticle and non-stoichiometric PbTiO_3_ (Pb-NPTO) nanomaterial coexisted. In this Pb-NPTO nanostructure, The Pb nanocrystals were observed to form and grow within the PTO matrix by electron bombardment, which enhances the growth of nanocrystals. As shown in Fig. 2 and Fig. S2 in the Supplementary Information, the PTO nanoparticles were exposed under electron beam with current density (J = 2.02 × 10^7^ A/m^2^). The size of Pb nanoparticles ranges from 4 nm to 15 nm which are observed throughout this study. We examined various PTO nanoparticles exposed under EBI to investigate the structural change and evolution of Pb nanocrystals.

In Fig. 2, HRTEM image evidently illustrates about Pb-NPTO structure. Fast Fourier transform (FFT) patterns of two different selected areas are shown in Fig. 2, namely 1 and 2. The bright spots in the pattern 1 can be well indexed to the ($1\bar{1}1$), (002) and ($\bar{1}11$) planes of cubic Pb, which belongs to the *Fm* $\bar{3}$ *m* (No. 225) space group and the bright spots in the pattern 2 can be well indexed to the (001) plane of tetragonal PTO. The lattice fringe spacing suggests that this particle is the tetragonal PTO, which belongs to the *P4mm* (No. 99) space group and has a lattice parameter of c = 4.156 Å and a = 3.905 Å. The outcome of *in situ* HRTEM study furnishes the formation, growth and coagulation of Pb nanocrystals. The nucleation and growth of a nanoparticle are shown as a function of time in the series of images presented in Fig. S3 (See Supplementary Information). It shows the existence of a 4–8 nm breadth of Pb nanoparticles at the circumference of the NPTO nanoparticle.

Initially, no Pb nanoclusters/crystals are observed on the PTO nanoparticle with the size of 30 nm. Increasing the EBI time to a critical value of 20 sec (with a 300 KeV electron beam, J = 2.02 × 10^7^ A/m^2^) resulted in the formation of many sphere like particles appearing on the PTO nanoparticle (indicated by the arrow). The number and the size of newly formed Pb nanoparticle increase with increasing the irradiation time. This newly formed nanoparticle is about 4 nm wide. After 40 sec, the width of the nanoparticle is increased to 7 nm which is about twice the width of the individual Pb nanoparticle. Prolonged irradiations on the sample affect the nature of the material. To elucidate the possible electron beam effect on the structure, we have recorded *in situ* TEM image of two different PTO nanoparticles under long periods of EBI (Movie S1 and Movie S2 in the Supplementary Information).

**Figure 3:**
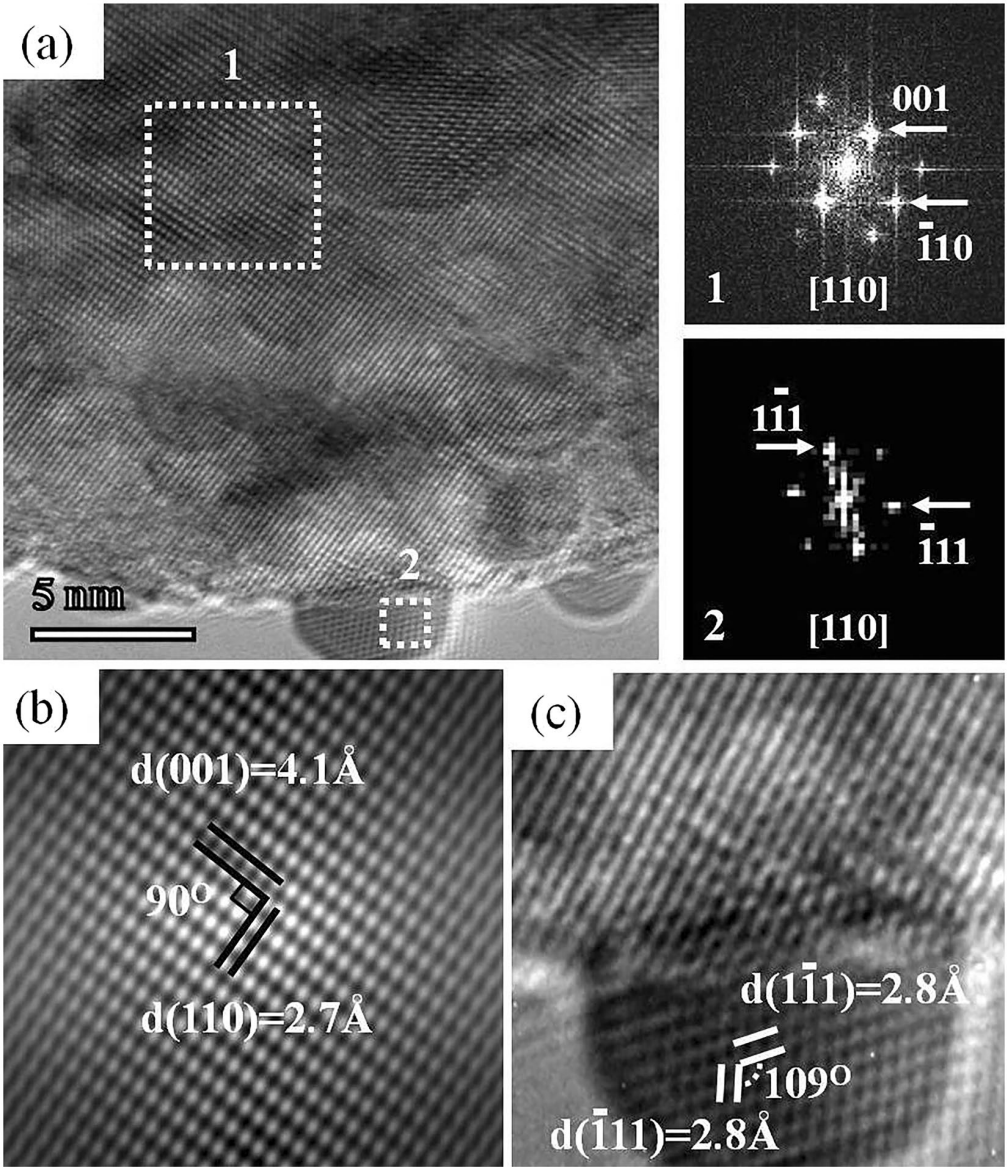
(**a**) TEM image of Pb-PTO nanomaterial. The right side image 1 and 2 are corresponding FFT patterns of PTO and Pb respectively, the selected area shown in Fig (**a**) by dotted rectangles. (**b**) FFT filtered image of the selected area as denoted 1 in Fig (**a**). (**c**) FFT filtered image of the selected area around Pb nanocrystal.

An extensive experimental result shows that pure Pb nanoparticles have different morphologies such as single crystal, twinned and multiple twinned. It is found that the Pb nanocrystals show structural fluctuation under intense electron-beam irradiation as other metal nanocrystals like gold, Indium, platinum, palladium and etc^27, 28, 43^. A single crystal of lead (Pb) in the size of 4 nm is observed in Fig. 3(a) along with PTO nanoparticle, which is exposed in EBI. The fast Fourier transformation (FFT) processing patterns of two areas in Fig. 3(a), shown as 1 and 2 on the right side of the Fig. 3(a), belong to PTO and Pb crystals, respectively. It is found that the bright spots in the pattern 1 can be well indexed to the (001), ($\bar{1}10$) and ($\bar{1}11$) planes of tetragonal PTO and also the pattern 2 can be well indexed to the ($1\bar{1}1$), ($\bar{1}11$) and (002) planes of cubic Pb viewed along [110] zone axis. The calculated spacing of the lattice fringes of the nanoparticle in Fig. 3(b) is about 4.1 Å and 2.7 Å, which is corresponding to the PTO crystal planes (100) and (110), respectively. Moreover, the spacing of the lattice fringes of the nanoparticle d_(111)_ = 2.8 Å in Fig. 3(c) belongs to the pure Pb in face-centered-cubic (fcc) structure with a lattice constant 4.9 Å. These results validated that the nanoparticles are a mixture of both pure Pb and PbTiO_3_ coexisting in the same environment. Note that, oxide or other impurities were not observed on the surface of the nanoparticle. To confirm this we looked for other oxides i.e. PbO_2_, Pb_2_O_3_, TiO_2_, etc. However, these oxides were not presented in this system. To elucidate the TEM image of the Fig. 3(b) and (c), we compared the simulated HRTEM image and the obtained one from experiment. Figure S4 in the supplementary Information shows the simulated HRTEM images, which is consist with the PTO and Pb material.

**Figure 4:**
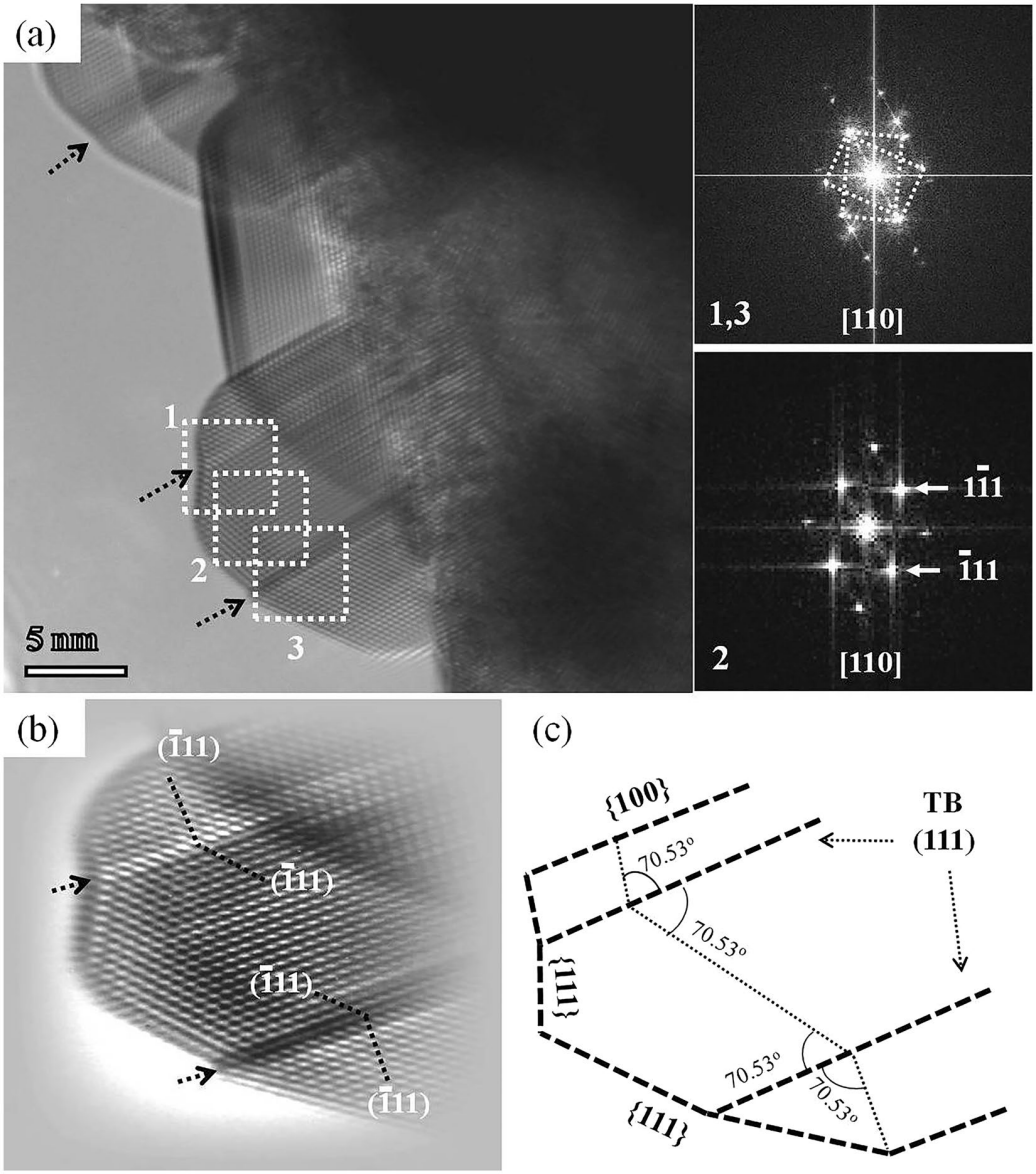
(**a**) HRTEM image of twinned Pb-PTO nanomaterial in [110] zone axis. The right side images 1, 3 and 2 are corresponding FFT pattern of twinned Pb, the selected area shown in Fig. 4 (a) by dotted rectangles. (**b**) FFT image of the twinned Pb nanocrystal in Fig (**a**). (**c**) Schematic diagram of the twinned Pb nanocrystal.

Twinned structures are regularly observed in the fcc structured metal nanoparticles^48–51^. Twin boundaries (TBs) are coherent planar defects where atom arrangements on one side are mirror reflections of those on the other side. In fcc metals, low energy twin planes are of {111} type that enclose an angle of 70.53°. Figure 4(a) shows a HRTEM image of the twinned Pb nanoparticles on the PTO matrix. The right side images are the corresponding FFT pattern of the selected areas, namely 1, 2 and 3 in the Fig. 4(a). The 1 and 3 selected area in the Fig. 4(a) are on the twin structures, 2 is off the twin structure. The FFT pattern of 1 and 3 is similar and the bright spot can be indexed as the ($1\bar{1}1$), ($\bar{1}11$) and (002) planes of cubic Pb viewed along [110] zone axis. The spots from twin structure are vividly observed in the FFT pattern and depicted by rectangles. Figure 4(b) is the Fourier filtered image, clearly shown the twin boundaries. The twin boundaries are denoted by black arrow in Fig. 4(a,b). There are two twin boundaries observed in the single Pb nanoparticles, which appeared as zigzag shape. In addition, we observed the multiple twinned Pb structure, which is illustrated in Fig. S5 (See Supplementary Information).

To make sure that Pb-NPTO nanocomposites are produced by exposure to the electron beam and not by rapid localized specimen heating followed by specimen melting process, the electron beam first focused on to arbitrary area and after that shifted and focused on various PTO nanoparticles that were not previously exposed to the e-beam. As shown in obtained images are various Pb-NPTO nanocomposite structures obtained from different precursor. In the process, The Pb nanoparticle population increased with EBI time, we discovered that the population as well as the size of Pb nanoparticles remains constant, when the irradiation is suspended. To affirm that we took a few unfocused TEM images, which are not greatly exposed to the EBI (Fig. S6 in the Supplementary Information). While in deliberated Pb nanocrystal structural study, the e-beam was highly focused on the particular Pb nanocrystal and it exhibits structural fluctuation other than nearby Pb nanocrystals. It demonstrates that pure metallic characteristic Pb nanocrystal is coexisted with NPTO nanoparticle. To the best of our knowledge, this novel nanocomposite has seldom been observed in perovskite material under EBI in real time observation.

### X-Ray Energy Dispersive Spectroscopy (EDS) analysis

**Figure 5:**
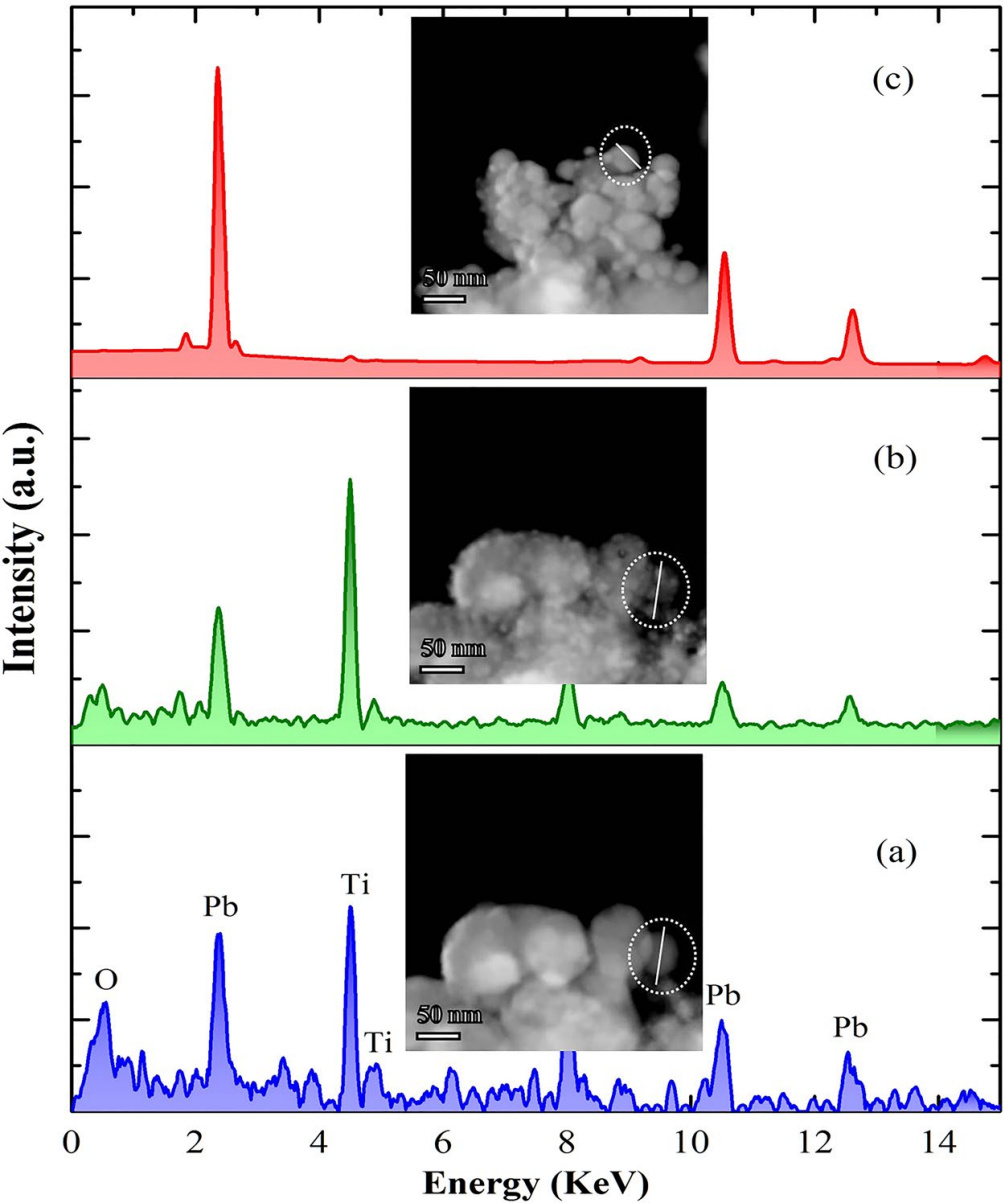
(**a**,**b** and **c**) are EDS spectrum acquired from the PbTiO_3_ sample before, after 80 sec and prolonged point of irradiation, respectively. The insets are corresponding High angle annular dark field (HAADF) images of the sample, which are analyzed by EDS method. The circle denoted a line profile scanned region.

In order to study the changes in chemical composition of the material, we exploited EDS method at different stages during the irradiation process. Figure 5 shows the typical EDS spectrum at different stages of irradiation. The spectrum acquired at the beginning of the experiments shows Pb, Ti and O peaks (inclusive of Cu and C peaks which belong to the sample grid). At this point, the semi-quantitative analysis was consistent with the atomic composition with the PbTiO_3_. After sample irradiated under e-beam, the EDS spectrum of the sample shows that the oxygen to Pb/Ti ratio decreased significantly, which argues that the particles consist of non-stoichiometric PbTiO_3_. Prolonged irradiation decomposed a PTO nanoparticle entirely. Consequently Pb nanoballs are formed around the amorphous matrix, which can be viewed in the EDS spectrum.

Figure S7 in the Supplementary Information illustrates TEM and HAADF-EDS spectrum elemental mapping images of the PTO nanoparticle before and after irradiation in identical region. The obtained data for different Pb, Ti and O concentrations revealed an excess of Pb with a deficiency in oxygen. The deficiency in O due to electron-stimulated desorption process (ESD) followed by oxygen lost in the vacuum. These results supported that the volatilization of Pb does take place in this process, as an alternative it forms a novel Pb supported Pb_1-x_TiO_3-y_ system. At the initial point, the clear PTO nanoparticle were seen in the TEM image, after irradiated by electron beam the material morphology was turned into fuzzy cloud appearance due to the arrangement of small Pb nanoballs on the surface of the sample. To derive the exact chemical formula, we calculated the atomic percent (at. %) value from elemental mapping spectrum at the different area of scanned region. The values and scanned region are bestowed in detail in the Supplementary Information.

### X-Ray Photoelectron Spectroscopy (XPS) analysis

**Figure 6:**
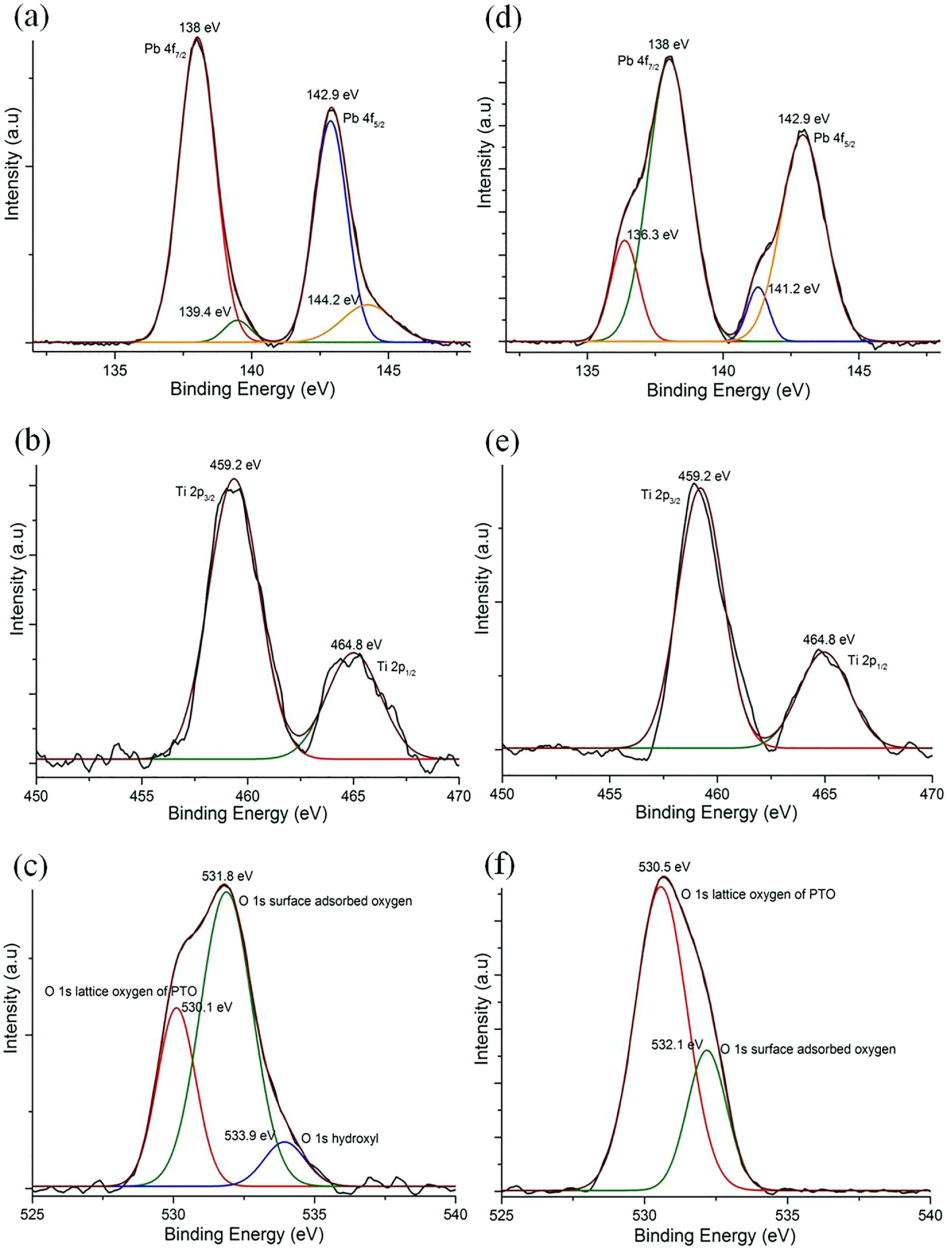
(**a**–**c** **)** XPS spectra of non-irradiated PTO nanoparticle. (**d**–**f**) XPS Spectra of irradiated PTO nanoparticle. From top to bottom core level spectra of Pb 4f, Ti 2p and O 1s plotted with deconvoluted spectra.

Surface features and electronic band structures of non-irradiated and irradiated PTO nanomaterials were examined using XPS and Gaussian fitting. The XPS survey spectra of the two samples are shown in Fig. S10 (Supplementary Information). As expected, the spectrum of the non-irradiated PTO shows higher Pb than observed in the irradiated PTO. It can be seen that the materials consists of Pb, Ti and O elements along with C 1s peak. Figure 6(a–c) shows narrow scan XPS spectra of Pb 4f, Ti 2p and O 1s, respectively, of a non-irradiated PTO sample. The Pb 4f spectra can be deconvoluted into two major and two minor components of Pb 4f_7/2_ and Pb 4f_5/2_ as observed in Fig. 6(a). The major peaks at 138 and 142.9 eV are attributed to lead lattice in PTO crystal. The minor peaks at 139.4 and 144.2 eV may arise due to the formation of negligible amount of air species)^52^. The spin-orbit splitting (Δ (4 f_7/2_-4f_5/2_)) for Pb 4f is 4.9 eV, which is consist with the PTO nanowire value^53^. Ti 2p deconvoluted spectrum shows only one spin orbit doublet as shown in Fig. 6(b). The first component around 459.2 eV is attributed to Ti 2p_3/2_ peak, whereas the second one around 464.8 eV is attributed to Ti 2p_1/2_ peak, which are larger than those reported for the PTO ceramics^54^. The difference between Ti 2p_3/2_ and Ti 2p_1/2_ spin-orbit splitting is 5.6 eV, which is well matched with the earlier reported ferroelectric materials with Ti atom in TiO6 octahedron^55^. The O 1s spectrum can be deconvoluted into three components as shown in Fig. 6(c). The major peak of higher binding energy 531.8 eV is assigned to adsorbed oxygen, the second major component at low binding energy 530.1 eV is assigned to lattice oxygen in the PTO. Figure 6(d–f) shows core-level XPS spectra of Pb 4f, Ti 2p and O 1s, respectively, of an irradiated PTO sample. Considering Pb 4f and O 1s spectra, the irradiated sample shows significant alterations in its surface characteristic with its counterpart non-irradiated PTO. It’s clearly seen that Pb 4f doublet exhibits two Pb 4f_7/2_ components at 136.3 eV and 138 eV. The peak at 138 eV is attributed to Pb at perovskite lattice and its intensity is lower than the non-irradiated Pb 4f spectrum. The lower binding energy components at 136.3 eV is assigned to Pb metal. The calculated downward shift binding energy is 1.7 eV. This indicates that the Pb metal co-exists with the non-stoichiometric PTO. As we discussed earlier, the Pb metal formed by the reduction of Pb^2+^ ions to Pb°. T J Zhu *et al*.^55^ reported the same phenomenon when the sample was sputtered by Ar^+^ ion. Moreover, in Fig. 6 (e) O 1s spectrum of irradiated sample shows two components at 530.5 and 532.1 eV. In contrast, we did not observe any significance difference between Ti 2p spectrum of non-irradiated and irradiated PTO samples as shown in Fig. 6(f).

**Figure 7:**
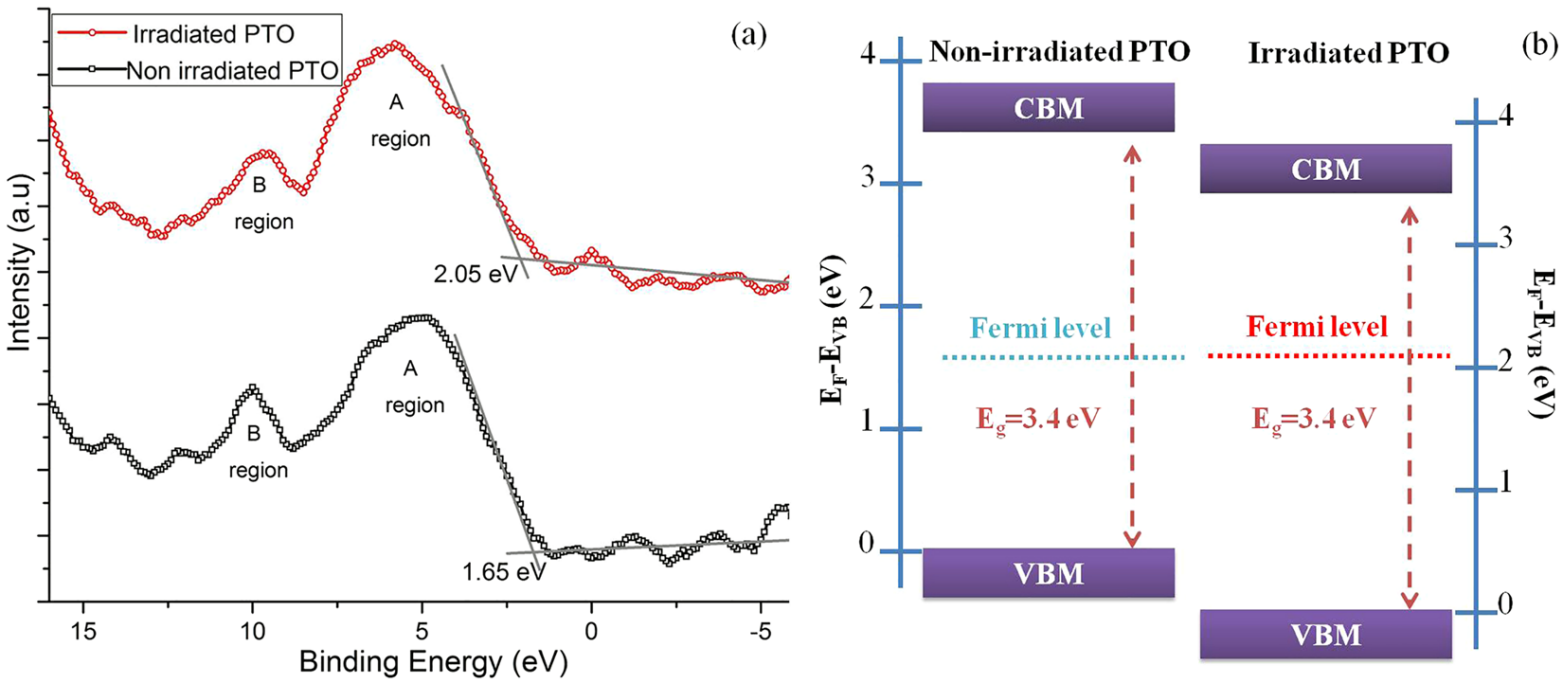
(**a**) valence band spectra of non-irradiated and irradiated PTO nanoparticle. (**b**) Band alignment structure of the non-irradiated PTO and irradiated PTO.

As a consequence of electron bombardment new electronic states appear in the bulk band gap and the structure of the valence band is changed. Figure 7(a) shows valence band spectrum of the non-irradiated and irradiated PTO. For the non-irradiated PTO, The band A region consists basically of O 2p states hybridized with Pb 6s, Pb 6p and Ti 3d states, this region is the evidence for the Pb-O-Ti bonding interaction. The total width of the region is approximately 6 eV. The band B region is assigned to mainly Pb-O boding interaction. A.T Kozakov *et al*.^56^ demonstrated that the B region was diminished when the Pb content becomes to zero in Pb_1-x_Ba_x_TiO_3_ materials. The irradiated PTO valence band structure shows significant changes in the region of A and B band. The total width of A region is 5.7 eV. The intensity of the B region reduces due to the diminishment of the Pb-O bond interaction. As shown in Fig. 7, by taking the baseline intercept of a linear fit to the valence band edge, the valence band maximum (VBM) energy positions in the non-irradiated and irradiated PTO were determined to be 1.65 ± 0.1 eV and 2.05 ± 0.1 eV, respectively. The change in VBM position can be understood by the theory of metal-semiconductor contact. Before irradiation, the VBM of PTO nanoparticle is 1.65 ± 0.1 eV, which indicates that PTO nanoparticle is weak p-type (the band gap of PTO is 3.4 eV). After irradiation, the newly formed metallic Pb nanoparticles contact with PTO nanoparticle, changing the Fermi level and thus VBM^57^. The Schottky limit of the valence band offset can be determined as 2.6 eV, from the work function of Pb (4.3 eV), and the electron affinity and band gap of PTO (3.5 and 3.4 eV). The Bardeen limit of valence band offset for PTO (1.95 eV) has been calculated by Robertson, assuming the metal-induced gap states model. Our experimentally determined VBM of Pb/PTO located at 2.05 ± 0.1 eV below the Fermi level, which is between the two limits and more close to the Bardeen limit. The limits of Fermi level variation study of the PbTiO_3_/SrTiO_3_ interface reveals that the Fermi level increase with the increase of STO thickness on the PTO^58^. The deposition of STO modified the Pb 6s electron contribution in the VBM of PTO. Surprisingly, we observed the same phenomenon in the Pb metal nanoparticle embedded in the Pb_1-x_TiO_3-y_ nanomaterial.

## Discussion

Electron radiation damage of materials in the TEM study is commonly observed phenomenon^59^. The lead titanate nanoparticle transforms into pure lead non-stoichiometric lead titanate (Pb-NPTO) nanocomposite system under electron irradiation. The bright field images and corresponding FFT pattern clearly show the low stability of the PTO and the induced reduction of Pb^2+^ ions by electron irradiation. The coexistence of pure Pb and NPTO is observed throughout this study. Though, there are many literatures discussed on the electron–materials interaction, each system has to deal with particular concern. The absolute mechanism of formation of the Pb-NPTO system by electron irradiation is still not understood. We provide the feasible mechanism of this phenomenon. When an electron interacts with the matter at high momentum, there are two possibilities of the mechanism involved i.e. elastic scattering and inelastic scattering. Both alter the nature of the materials by electrostatic charging, atomic displacement, specimen heating, mass loss and radiolysis process. We believe that electron beam sputtering or ionization of PbTiO_3_ occurs in this study. To deduce the formation mechanism of the Pb-NPTO system, we resolve (a) atomic displacement and radiolysis, (b) specimen heating processes caused by incident electrons.

Atomic displacement and radiolysis: Atomic displacement will occur when the incident electron energy (E_0_) exceeds the displacement energy of the materials (which is depending on the nature of the specimen-thickness, binding energy, cohesive energy and etc) at high angle elastic scattering. The energy transferred from the incident electron to the atomic nucleus is given by^60–62^.

1 $$E={E}_{max}si{n}^{2}(\theta /2)$$

2 $${E}_{max}=[2\,{E}_{0}({E}_{0}+2{m}_{e}{c}^{2})]/M\,{c}^{2}$$

Where θ is the deflected angle of the electron in the field of atom nucleus, E_0_ and E_max_ are the incident electron energy and maximum energy in eV (which is transferred at θ = 180°) respectively, m_e_ and M are the mass of the electron and the nucleus in Kg, respectively, c is the speed of the light in m/s. The maximum energy transferred from the 300 KeV electron beam to Pb, Ti and O are 4.3 eV, 18.8 eV and 56.4 eV, respectively, which are calculated from the equation (2), such high energies are able to generate the atomic displacement and also mass transfer in the material. R F Egerton *et al*.^60^ showed that the electron beam sputtering could occur by 200 KeV electrons for light and medium-Z atoms (including Au and Pb elements). Hence the sputtering process mainly involves low-Z atom oxygen in our case due to high E_max_ value. However, in insulating material like PbTiO_3_ the knock-on displacement process is quenched due to the high density of the delocalized electron (while favourable in the conducting material). Withal, we observed the Pb and O atom displacement in this system. To shed more light on this growth mechanism, we considered the radiolysis process too (which occurred by electron beam degradation through inelastic scattering).

The incident electron beam breaks the bond between Pb-O rather than Ti-O, which is located on the surface of the PbTiO_3_ structure and longer, since the required threshold energy of surface atomic displacement is lower than the interstitial site (Ti) atomic displacement. Here Pb^2+^ ions are reduced to Pb and the cation vacancy can be obtained in the crystal. Continuous irradiation affects the structure and leads to more bond breaking and produces the Pb on the surface of the degraded material. The number of Pb nanoparticles increases with the time of the radiation. Usually the cation vacancy occurs with an oxygen vacancy (Schottky defects) and tends to form the pair of cation and oxygen compounds like PbO, PbO_2_, Pb_2_O_3_ but we observed only pure Pb metal nanoparticle on the surface. And also, neither Ti-O bond breaking nor newly formed Ti_x_O_y_ like compound were observed, indicating that desorption of the oxygen atom takes place and the oxygen lost into the vacuum. These results are highly consistent with the EDS and XPS analysis. Earlier studies have been proven that the electron-stimulated desorption occurs in transition-metal oxides (such as WO_3_, TiO_2_, LiNbO_3_) via Knotek-Feibelmen (KF) mechanism. Agreeing to the KF mechanism, the core hole/inner-shell vacancy created on the metal ion (Pb^2+^) by the incident electron is followed by (interatomic) Auger decay from the oxygen^34^. The mechanism expressed by following equation^46^.

3 $${{\rm{PbTiO}}}_{3}\to {\rm{xPb}}+{{\rm{Pb}}}_{1-{\rm{x}}}{{\rm{TiO}}}_{3-{\rm{y}}}+{{\rm{yV}}}_{{\rm{O}}}+{y/\mathrm{2O}}_{2}$$

4 $${{\rm{yV}}}_{{\rm{O}}}\to {{{\rm{yV}}}_{{\rm{O}}}}^{\ast \ast }+2{{\rm{ye}}}^{-}$$

**Figure 8:**
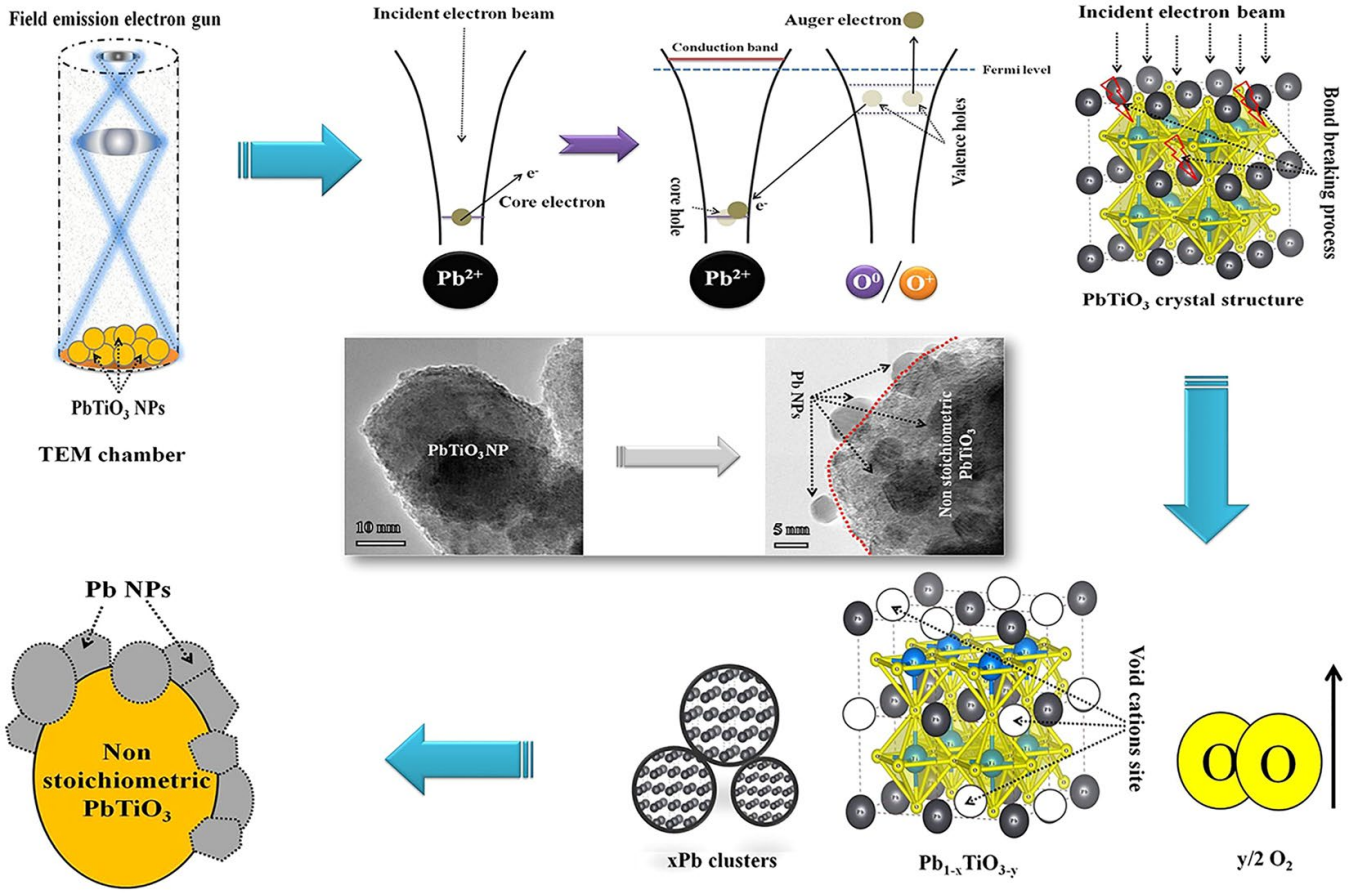
A schematic expression of formation of xPb-Pb_1-x_TiO_3-y_ via Knotek-Feibelmen (KF) mechanism.

Where V_O_ is the oxygen vacancy, Our deliberate study of structural analysis of this system revealed that the pure Pb and non-stoichiometric PbTiO_3_ coexisted as well as the Pb metal nanoparticles show structural fluctuation under electron irradiation as like other metal nanoparticles. In general notation the fabricated material via electron beam irradiation is xPb-Pb_1-x_TiO_3-y_. Figure 8 provides the schematic idea of the xPb-Pb_1-x_TiO_3-y_ material formation.

Specimen heating effect: Consideration of beam heating effect, the energy lost by the electron (when it interacts with the material) in the form of thermal energy results in the increase of temperature. We calculated the beam induced rise in temperature with respect to the time by L.C. Liu *et al*. proposed model^36^. Figure S11 in the Supplementary Information shows rapid increase in temperature of PTO during initial stage of electron beam irradiation (15.6 K increased at 1 sec); further electron irradiation results in only slight increase in temperature (17.9 K at 200 sec). The result clearly states that the increase of temperature is very low. R. F Egerton *et al*. reported that despite the fact that the high current density involves a very small beam diameter, the temperature rise is usually insignificant (in our case the beam diameter is 50 nm). Therefore the beam induced specimen heating affects less the melting of the PTO nanomaterial. Melting temperature of 30 nm size spherical PbTiO_3_ nanoparticle is ≈1500 K (bulk melting temperature is 1554 K). The detailed calculation is bestowed in the supplementary information section.

When an e-beam illuminates the precursor, the electron-nucleus interaction leads to the ionization of few atoms in the specimen due to its insulating nature. Consequently, the electric field increases in high level at the edge of the illuminated area due to poor conductivity of the PbTiO_3_ nanoparticle. This leads to the ejection of the metallic Pb atom from the precursor. Newly emerged Pb atom grown as Pb nanocrystal, which is formed by nucleation, coalescence and Oswald ripening process. A growth mechanism was proposed to explain the experimental observation and was presented in Fig. S14 (See Supplementary Information). Xing Peng et al.^26^ discovered that the Pb^2+^ cation deficiency is the important candidate as negative thermal expansion materials. Additionally the Pb nanoparticles are superparamagnetic in nature. In our study, we designed the superparamagnetic nature Pb nanocrystals embedded in cation deficient ferroelectric PbTiO_3_ nanoparticle. Blending of these two functional properties in xPb-Pb_1-x_TiO_3-y_ nanocomposite material leads to open the pathway for new generation multifunctional system. A more detailed study necessitates explaining the electron-perovskite materials interaction and its non-stoichiometric counterpart formation with respect to beam current, thickness of the materials and etc. Nevertheless the fabrication of Pb-NPbTiO_3_ (xPb-Pb_1-x_TiO_3-y_) cation vacant perovskite material via electron beam irradiation in TEM is of great significance for the functional material development and application.

A novel nanocomposite material xPb-Pb_1-x_TiO_3-y_ is successfully fabricated in simplest technique and it is proven that the proportions in the composite material can be altered by e-beam irradiation. *In situ* HRTEM study on 300 KeV electron beam stimulated pure Pb metal nanoparticles on PbTiO_3_ matrix has been investigated. The study reveals that the Pb nucleation and structural fluctuation are controlled by the irradiation time. In addition, the electronic structure and surface features were studied extensively by using the XPS for non-irradiated and irradiated PTO nanomaterials. The novel results reveal that the irradiated PTO exhibits n-type conductor feature, while the non-irradiated PTO exhibits weak p-type. The calculated valence band offset is in good agreement with the reported values. Moreover, the twin and multiple twinned structures are observed in pure Pb metal nanoparticles. The coagulation of Pb–Pb nanoparticle leads to different structures depending on the irradiation time. The sizes of the Pb nanoparticles are various with respect to the internal structure i.e single crystal Pb has a size of 4-6 nm, Twinned Pb has a size of 9-15 nm. The coexistence of this Pb nanocrystal attached to the cation deficient nanoparticle Pb_1-x_TiO_3-y_ perovskite material system is a prominent candidate for functional applications. The study of interaction of PbTiO_3_ nanoparticle and electron uncovered that not only the structural transformation occurred in the material and also it is a new approach to fabricate the novel composite system.

## Methods

### Synthesis of PbTiO_3_ nanomaterial

The lead titanate (PbTiO_3_) nanoparticles were synthesised by sol-gel method. The precursors used for preparing lead titanate (PT) sol were Lead acetate trihydrate [Pb(OCOCH_3_)_2_.3H_2_O], and Titanium IV butoxide [Ti(OC(CH_3_))_4_]. Acetic acid [CH_3_COOH] and acetylacetone [C_5_H_8_O_2_] utilized as a solvent and a chelating ligand, respectively. All chemicals are purchased from Sinophram chemical reagent Co.Ltd, China as AR grade. The Pb^2+^ solution is prepared by dissolving Lead acetate trihydrate in acetic acid. The solution is heated and stirred for 25 min. Then 10 ml of equal molar acetylacetone was added into it. Finally, titanium butoxide in stoichiometric ratio is slowly added into the solution with continuous stirring for 25 min to prepare PT sol. The PT sol has dried under in air atmosphere at 120 °C to remove organic residuals. Then, as synthesised powder sample goes through calcinations process. The powder was calcined in small batch (2 g) in silica crucible at 450 °C in air atmosphere for 1 h.

### Fabrication of Pb-NPTO nanomaterial

The average size of the nanoparticles is 25 nm (which is used in this study). The PbTiO_3_ nanoparticle TEM specimens were prepared by the brief agitation of synthesised material, which is dispersed in ethanol in an ultrasonic bath and dipped over a carbon-film copper grid and placed into the TEM. The sample was allowed to expose in electron beam and study the formation of Pb-NPTO nanomaterial. (Full details of *in situ* HRTEM and XPS study were bestowed in the supplementary Information).

## Electronic supplementary material

Ma-Supplementary Material (SREP-17-15562A)-Revised

Movie S1

Movie S2
