# Supplementary material for: Designing of metallic nanocrystals embedded in non-stoichiometric perovskite nanomaterial and its surface-electronic characteristics
Source: Sci Rep. 2017 Aug 21;7:8343. doi: 10.1038/s41598-017-09031-5 (PMC5567205; doi:10.1038/s41598-017-09031-5)
Supplement: Supplementary file 1 — Ma-Supplementary Material (SREP-17-15562A)-Revised [file 41598_2017_9031_MOESM1_ESM.pdf]

# Designing of metallic nanocrystals embedded in non-stoichiometric perovskite nanomaterial and its surface-electronic characteristics

Jagadeesh Suriyaprakash[1,2], Y. B. Xu[1], Y. L. Zhu[1], L. X. Yang[1], Y. L. Tang[1], Y. J. Wang[1], S. Li[1,2] and X. L. Ma[1,3]*

[1]*Shenyang National Laboratory for Materials Science, Institute of Metal Research, Chinese Academy of Sciences, 72 Wenhua Road, 110016, Shenyang, China.*

[2]*University of Chinese Academy of Sciences, 100039, Beijing, China.*

[3]*State Key Laboratory of Advanced Processing and Recycling of Non-ferrous Metals, Lanzhou University of Technology, 730050 Lanzhou, China*

*Correspondence should be addressed to X. L. Ma (xlma@imr.ac.cn)

**Supplementary Materials Include:**

Figures S1-S13

Table S1

Movies S1, S2.

## 1. XRD analysis

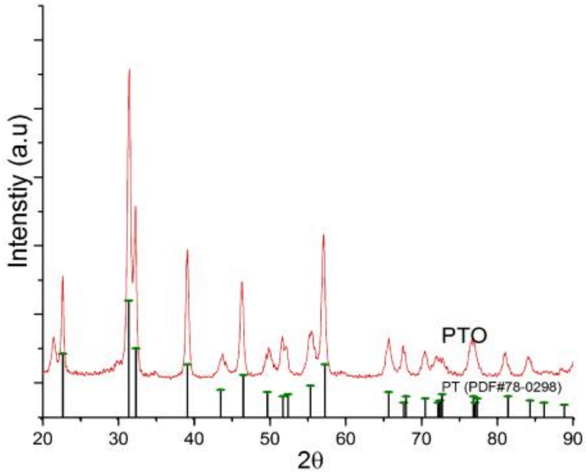


Figure S1. XRD patterns of $PbTiO_3$ powder calcined at $450^{O}C$ temperatures for 1h. The XRD pattern reveals reflection lines corresponding to the expected single phase. The pure $PbTiO_3$ perovskite phase was clearly observed, which is calcined ($450^{o}C$) at below the Curie temperature ($490^{o}C$) of the material. The crystal system was represented as tetragonal (space group: P4mm). All the peaks are well matched with the JCPDS PDF#78-0298 standard data card. There is no evidence of bulk remnant materials and impurities.

## 2. HRTEM analysis

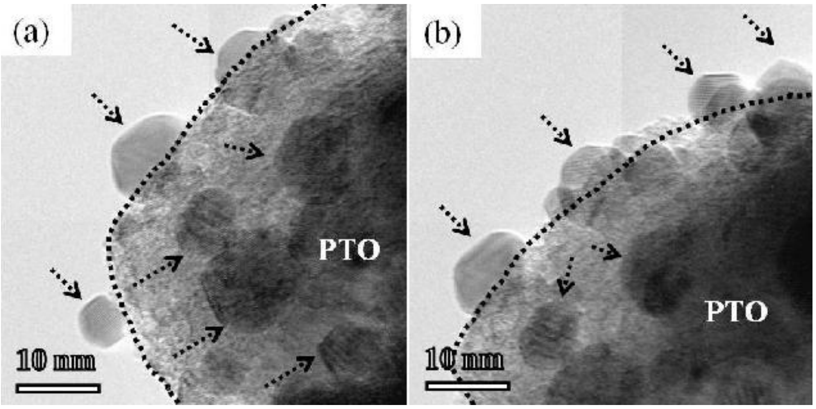


Figure S2. (a) and (b) TEM images showing two different face areas of Pb-NPTO nanomaterial after electron beam irradiation. The black dotted line depicts the boundary of the PTO nanoparticle. The arrow marks indicate the existence of Pb nanoparticle on PTO surface.

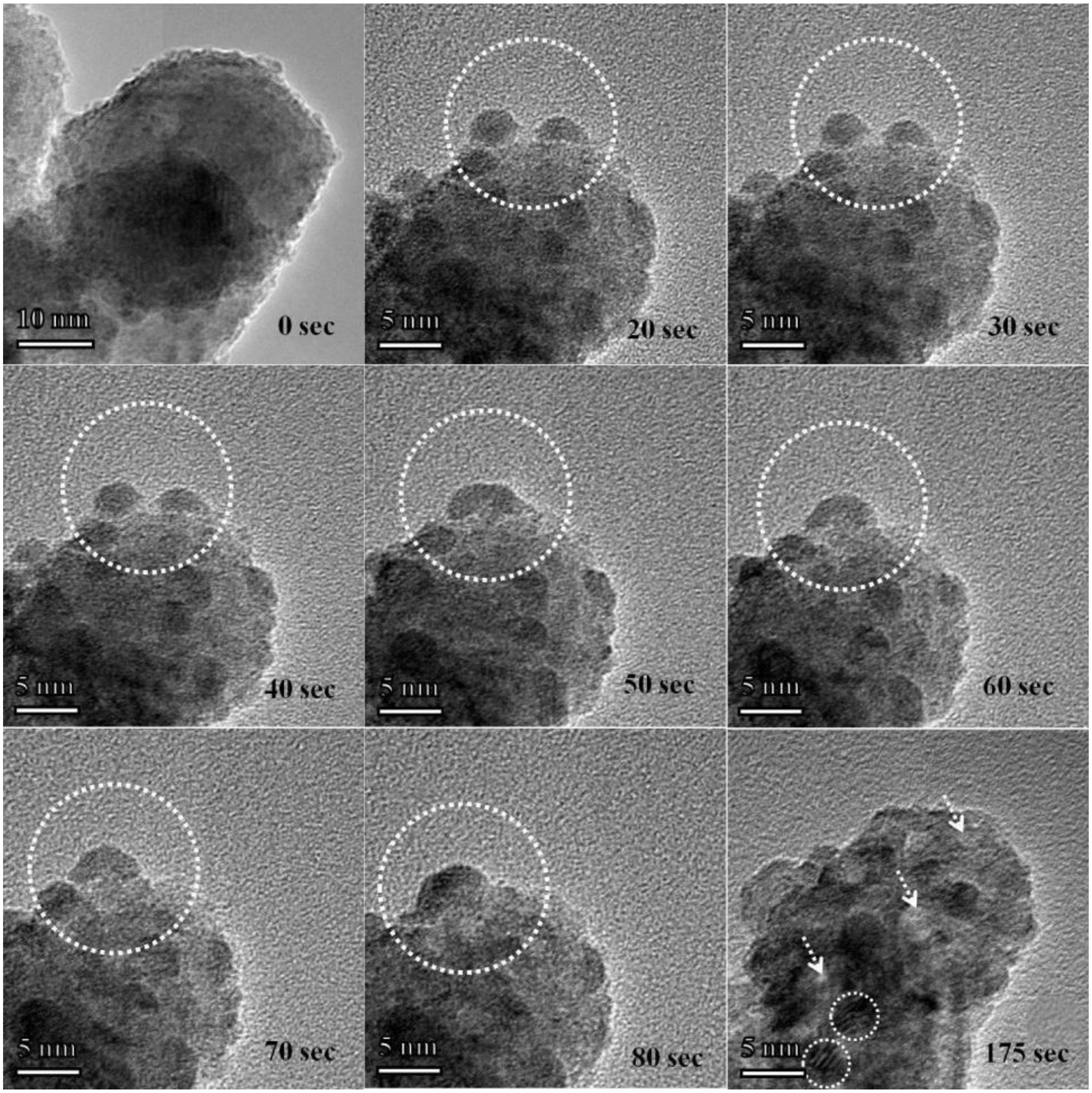


Figure S3. Time sequence series of images during electron irradiation on the sample. The circles indicate the coalescence of two Pb nanoparticles. The white arrows in the last figure indicate the void like space created in the material at 175 sec. The maximum size of the Pb nanoparticle observed in this study is 13 nm (Which has twinned structure). Doubling the time interval leads to increase in size as well as the population of the Pb nanoparticle till 80 seconds. After 80 sec, it creates structural damage (deformed into the void kind of structure).

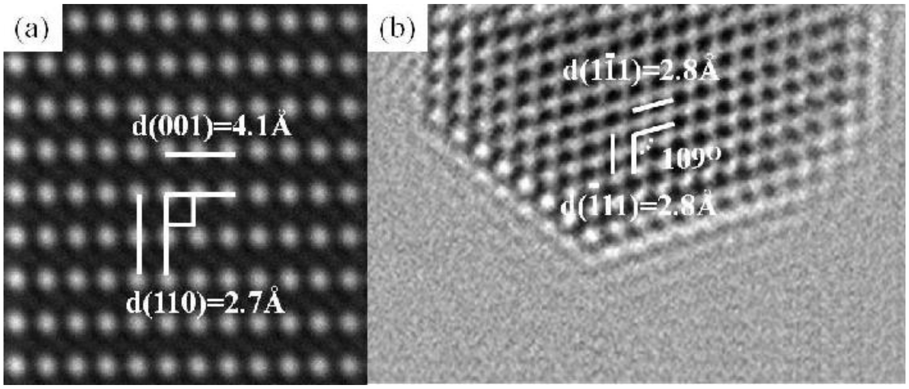


Figure S4. (a) and (b) simulated HRTEM image of PTO and Pb nanomaterial, respectively.

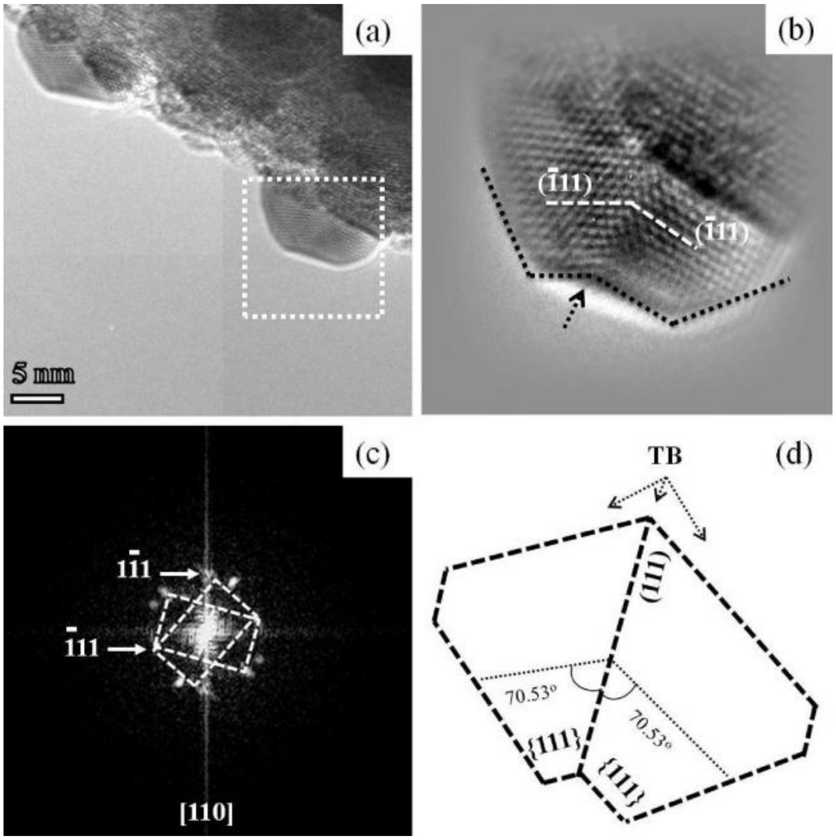


Figure S5 (a) HRTEM image of twinned Pb-PTO nanomaterial in [110] zone axis. (b) FFT image of the twinned Pb nanocrystal in Fig (a). (c) FFT pattern of selected are in (a). (d) Schematic diagram of the twinned Pb nanocrystal.

Cyclic twinned particles are more commonly observed in the fcc metal nanoparticles. The possible structures of multiple twinned metal particles are either decahedrons or icosahedrons. Sometimes both can be presented in the system under heavy electron irradiation. Fig. 8(a) shows a multiple twinned Pb nanoparticles on the surface of the PTO nanoparticle. Due to the electrostatic attraction and surface modification under the influence of the electron irradiation, the Pb nanoparticles are not isolated from the surface. So it's difficult to analysis the real structure of the Pb nanoparticle. The feasible explanation of the structure can be provided by based on the structural replicate on the other sides of the nanoparticle.

The Fourier filtered image of the single Pb nanoparticle of the selected area in Fig. 8(a) clearly indicates that the presence of twin boundary (which is denoted by arrow) and that exhibit slight grooving where they emerge to the Pb nanoparticle surface. The FFT pattern of the selected Pb nanoparticle revealed that the stronger reflection bright spots are correspond to the diffraction indices of ($1\bar{1}1$), ($\bar{1}11$) and (002) of the cubic structure of Pb, according to the JCPDS database (PDF#87-0663). Moreover, the twin nature, reflection observed and denoted by rectangles in Fig. 6(c). The schematic representation of the Pb nanoparticles and its twin boundaries are rendered in Fig. 6(d). The twin planes are separated by the angle $70.53^{o}$. As we mentioned earlier, the structural replication process on the other side of the nanoparticle leads to five fold multiple twinned particles as commonly observed in the fcc metal nanoparticle. Based on this assumption we derived that the Pb multiple twinned nanoparticle has decahedron shape. Normally the decahedron composed of the five tetrahedra. In this case, the non isolated particle had shown the two tetrahedra in the structure. The result consists with the earlier study of other fcc metals nanoparticle reported elsewhere.

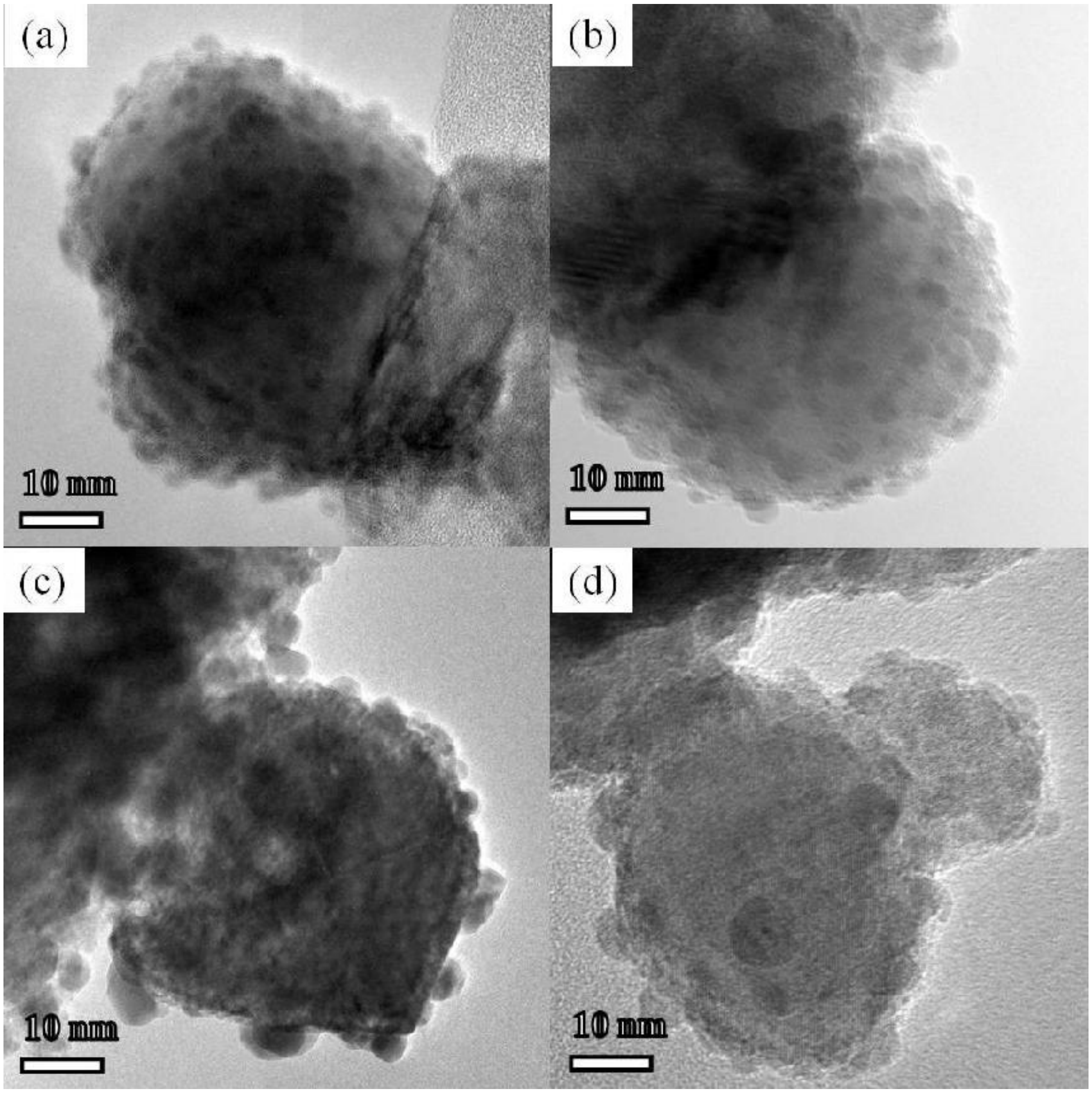


Figure S6. (a), (b), (c) and (d) are unfocused TEM images of the sample after irradiation suspended.

## 3. X-Ray Energy Dispersive Spectroscopy (EDS):

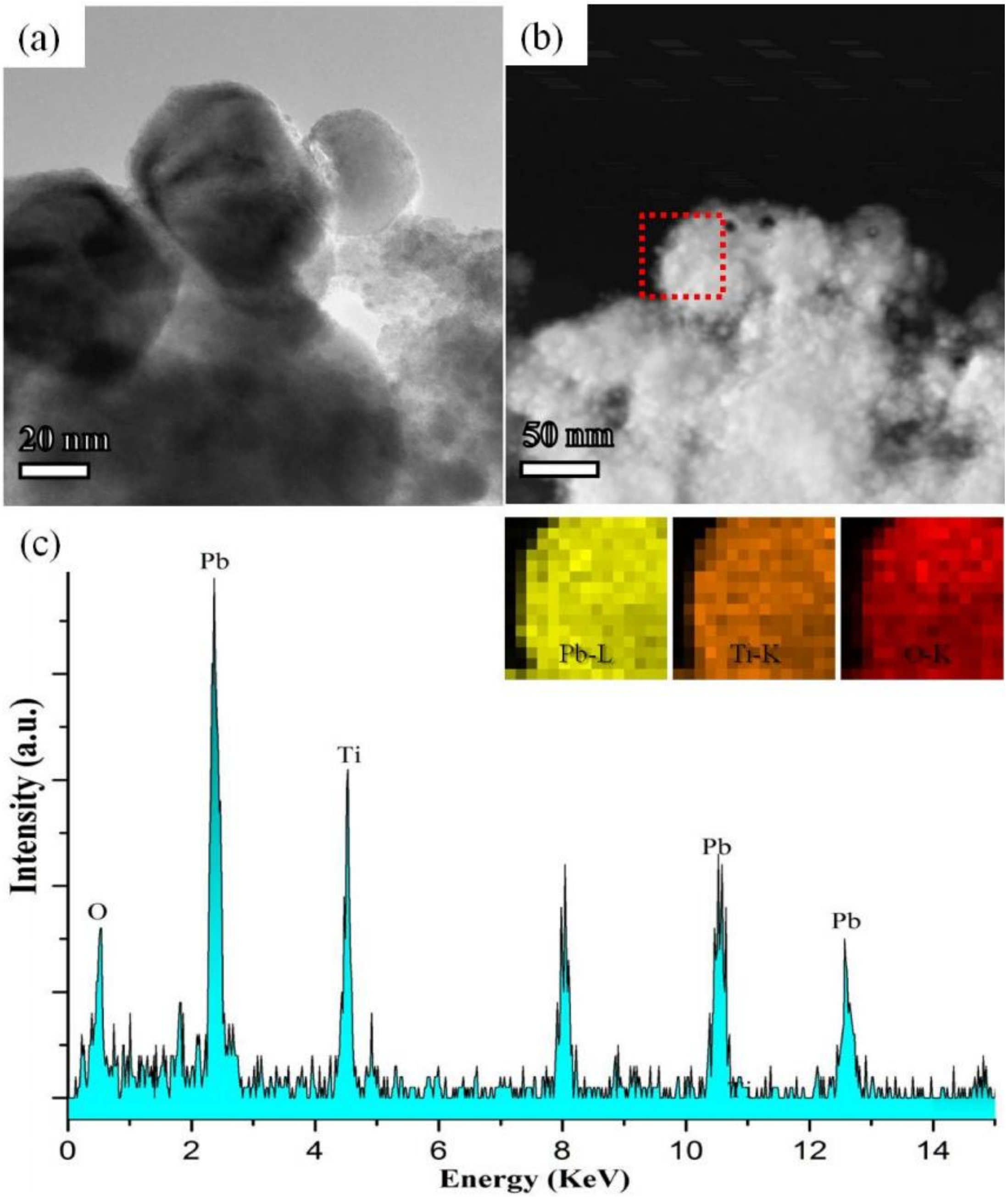


Figure S7. (a) TEM image of the PTO nanoparticle at initial point of irradiation. (b) HAADF image of the sample after irradiation, which is analyzed by EDS method. The rectangle denoted a scanned region. (c) EDS spectrum acquired from the $PbTiO_3$ sample after irradiation. The insets are atomic % chemical mapping images.

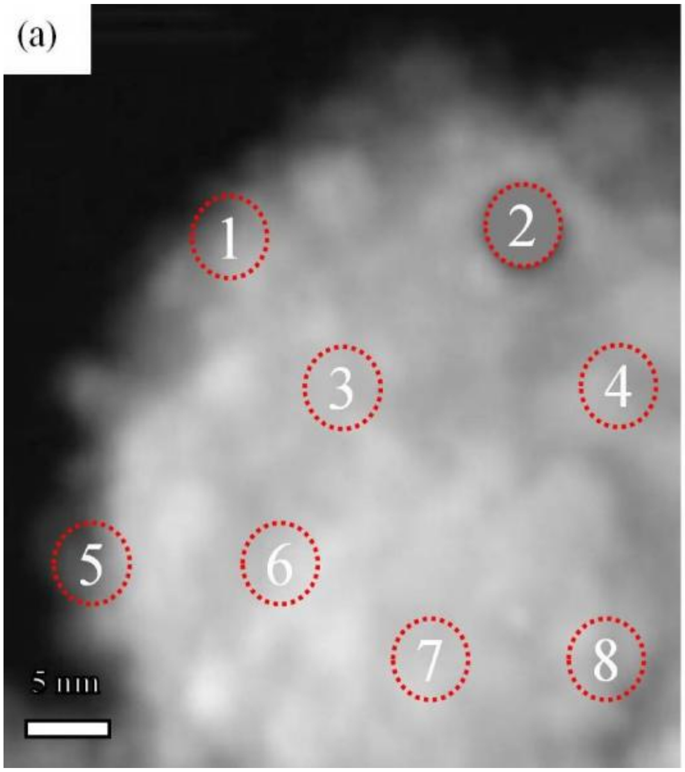


| Position | Pb at. % | Ti at. % | O at. % | Derived chemical formula |
|---|---|---|---|---|
| 1 | 36.21 | 20.43 | 43.35 | $Pb_{1.8}TiO_{2.2}$ |
| 2 | 33.65 | 20.58 | 45.76 | $Pb_{1.7}TiO_{2.3}$ |
| 3 | 20.62 | 19.79 | 59.58 | $PbTiO_3$ |
| 4 | 26.25 | 20.86 | 52.88 | $Pb_{1.3}TiO_{2.7}$ |
| 5 | 21.12 | 18.98 | 56.89 | $PbTiO_{2.7}$ |
| 6 | 32.64 | 20.71 | 46.64 | $Pb_{1.6}TiO_{2.3}$ |
| 7 | 30.78 | 20.68 | 48.53 | $Pb_{1.5}TiO_{2.4}$ |
| 8 | 29.26 | 22.11 | 48.62 | $Pb_{1.3}TiO_{2.7}$ |

Figure S8. (a) HAADF image of the sample after irradiation, the numbers are indicated the semi-quantification analysis carried out in different position and corresponding values are tabulated in the right hand side.

The derived chemical formulas in different positions illustrate that non stoichiometric PTO were obtained at most of the scanned region. From the calculated values we can clearly view the increase of Pb at.% and decrease of O at.%. It can be explained by the following three possible mechanisms.

1. $PbTiO_3 \longrightarrow PbO + TiO_2$
2. $PbTiO_3 \longrightarrow Pb_xO_y + Pb_{1-x}TiO_{3-y}$
3. $PbTiO_3 \longrightarrow xPb + Pb_{1-x}TiO_{3-y} + y/2\ O_2 \uparrow$

We well knew that the $PbTiO_3$ nanoparticles (PTONPs) will decompose while irradiated under electron beam. Agreeing to the step 1 and 2, PTO is decomposed into PbO, $TiO_2$, $Pb_xO_y$ and $Pb_{1-x}TiO_{3-y}$. If the formation mechanism complies with step-1 and 2, the semi quantification

EDS analysis must show the total at. % of Pb, Ti and O in scanned region which is either equal to $PbTiO_3$ or increase in Pb and O, but there is no evidence of such PbO / $TiO_2$ with $Pb_{1-x}$ $TiO_{3-y}$ formation. To be skeptical on PbO evolution from the PTO matrix, we deliberately analysed a newly emerged particle on the surface of the PTO matrix. The EDS spectrum shows that only Pb element presents at 99 at.% (Fig. 5(c)). Moreover the reduction of oxygen around 10 to 20 at.% is observed in this study. These results exhibit step-1 and 2 are not a promising mechanism. Considering step-3; after irradiation of PTONPs, the final products are Pb, $Pb_{1-x}$ $TiO_{3-y}$ and oxygen enters into vacuum as a gas, which is consistent with the EDS spectrum results. The deprivation of oxygen in the vacuum can be explained by an electron-stimulated desorption process. The increment of Pb at.% results in the formation of new Pb nanoparticles in the $Pb_{1-x}$ $TiO_{3-y}$ surface from another region. These particles will accumulate at random positions. When the element mapping has been performed on these positions, it turns out that a Pb/ $Pb_{1-x}$ $TiO_{3-y}$ mixture is newly formed. Therefore the derived chemical formula is $Pb_aTiO_b$ (where $a>1$ and $b<3$). On the whole, the step-3 mechanism is a possible way to explain the formation of pure lead supported in the cation deficient PTO matrix.

## 4. Electron beam parameters:

Table S1. Electron beam parameters of irradiation process

| S. No | Particulars | Value |
|---|---|---|
| **1** | Beam current | 39.7 nA |
| **2** | Beam radius | 50 nm |
| **3** | Current density | $2.02\times10^7$ A/m$^2$ |
| **4** | Energy of 300KeV electron | 318349.44 ev |

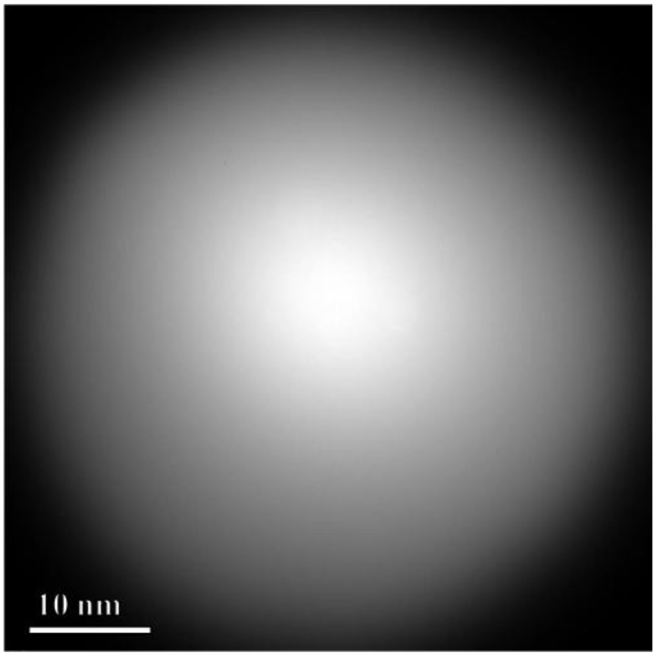


Figure S9. TEM image of electron beam spot, whose diameter is about 50 nm.

## 5. X-Ray Photo Electron Spectroscopy (XPS)

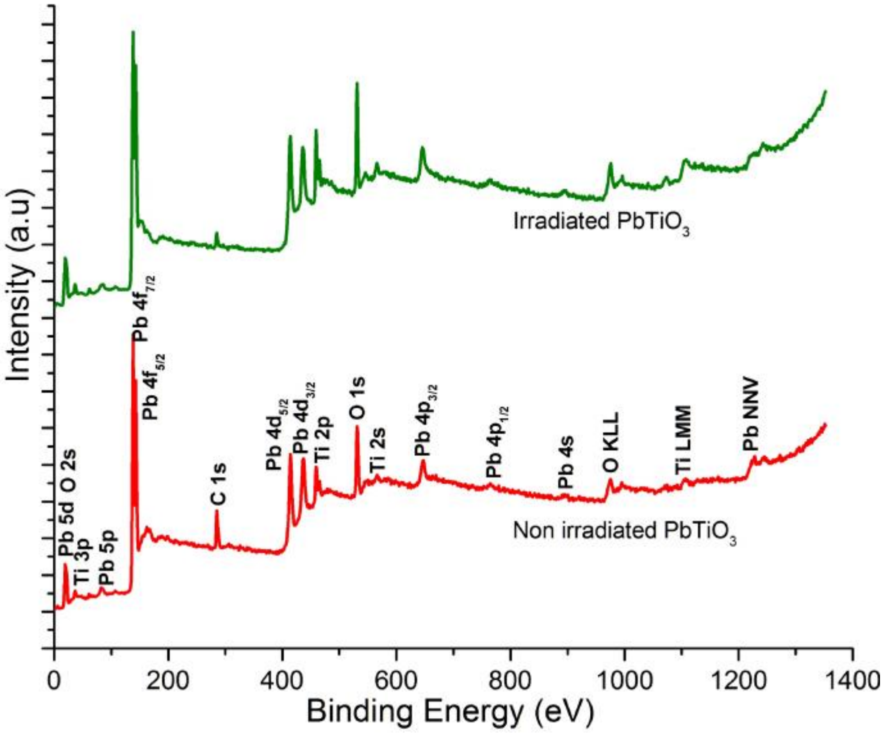


Figure S10. XPS survey spectra of both the non-irradiated and irradiated PTO

## 6. Specimen heating effect:

When a high energy of electron passes through the materials, they create the thermal spikes. The total energy loss of the electron,

$$Q = \frac{dE}{dx} = Q_e + Q_c$$

Where, $Q_e$ and $Q_c$ are electron excitation and coulomb encounter loss, respectively. These energy loses can be calculated by following equation,

$$Q_e = \Theta\left[\log\left(\frac{m^2\, c^4\ \ ^2}{Z^2\ I^2\ (1- \ \ ^2)}\right) - \ \ ^2 + 0.198\right] \qquad (1)$$

$$Q_c = \frac{\Theta}{1837.5A}\log\left(\frac{T_m}{T_a}\right) \qquad (2)$$

Where, constant $\Theta = 2\text{®}nZ^2 r_e^{\,2} mc^2 \big/ \ \ ^2$, $T_m$ = (560.8 / A) X (X+2) in eV, $T_a \approx$ (m/M) $(1+Z^{2/3})$ $R_h$, β=v/c=0.7760 for the case of $E_p$= 300 KeV, Z= 82 for Pb, $r_e$= $2.82\times10^{-15}$ m (classical electron radius), $R_h$ is Rydberg energy for hydrogen atom, A is the mass number of the atom, m and M is the mass of the nucleus involved in the interaction and mass of the electron respectively, Z is atomic number of nucleus, n is the number density in the particle, I is the average ionization of potential of the electrons in the atom.

The electron beam induced temperature increment ($\Delta T_e$) of PTO matrix for time $t_e$ seconds is given by,

$$\Delta T_e = \frac{3\,J\,Q}{8\,e\,c_v\ D\,d} R_e^{\,2} \log\left(1+\frac{4\,D\,t_e}{R_e^{\,2}}\right) \qquad (3)$$

Where D= $k_t/c_v$ d ($k_t$ is the thermal conductivity, $c_v$ is specific heat and d is the density), e is a charge of the electron, J is the current density, and $R_e$ is the effective beam radius. In our case, J= $2.02\times10^7$ A/m$^2$, $R_e$=25 nm. Using equ.(3) and values, we obtained the rise in temperature with respect to the irradiation time. We plotted $\Delta T_e$ vs $t_e$ curve shown in Fig. 3.

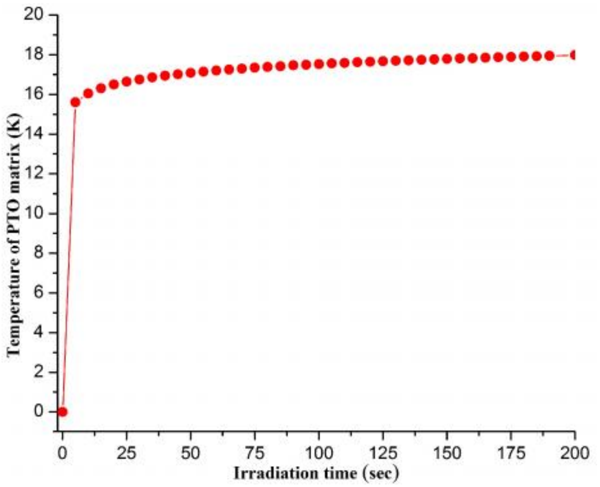


Figure S11. Calculated curve for rise in temperature of PTO matrix ($\Delta T_{e)}$ vs irradiation time ($t_e$).

Melting temperature of 30 nm size spherical $PbTiO_3$ nanoparticle is ≈1500 K (where the bulk meting temperature is 1554 K), which was calculated using following equation.

$$T_{mp} = T_{mb}\left(1 - \frac{N}{2n}\right) \tag{4}$$

Where, $T_{mp}$ and $T_{mb}$ are the melting temperature of the nanosolid and corresponding bulk material. N/n is a size and shape factor.

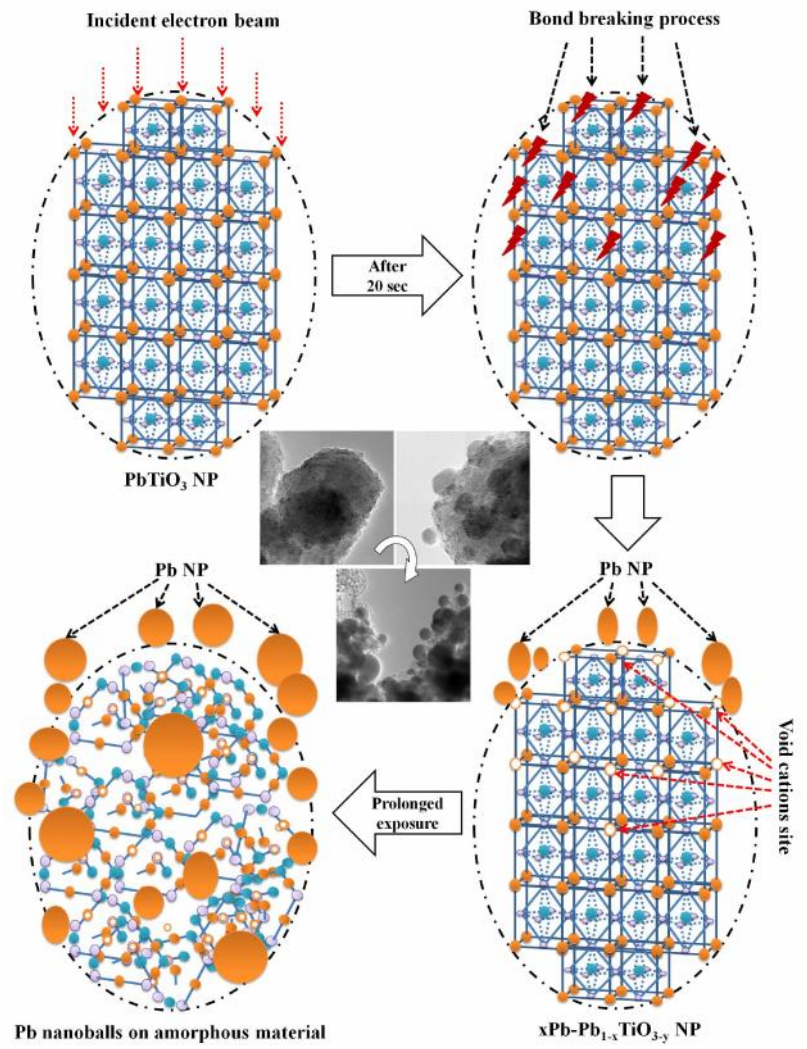


Figure S12. Illustration of the electron beam irradiation on the $PbTiO_3$ nanoparticle. After 20 sec of irradiation, the bond breaking process takes place and simultaneously Pb metal nanoparticle emerged on the surface of void $PbTiO_3$.

The phase transformation and the bonding with Pb clusters are considered to be the result of two effects of the electron beam. The first effect is oxygen atom displacement by electron via KF mechanism, and the second is localized Joule heating. The nucleation process involves Ostwald ripening and coalescence. Initially the fine particles form with the size of 4nm and then grow into a large crystal size of 7-13 nm. Once the particle growing bigger; the coalescence process starts, the small particles are diffused into one another. Neighbouring particles are agglomerate and merge together. The particles above 7 nm show the structural fluctuation and form a twin structure.

7. **Movies S1-S2:** The two different $PbTiO_3$ nanoparticles are illuminated by electron beam, recorded at 6 frames / sec, and played at 10 × speeds.

## 8. $Bi_4Ti_3O_{12}$ system

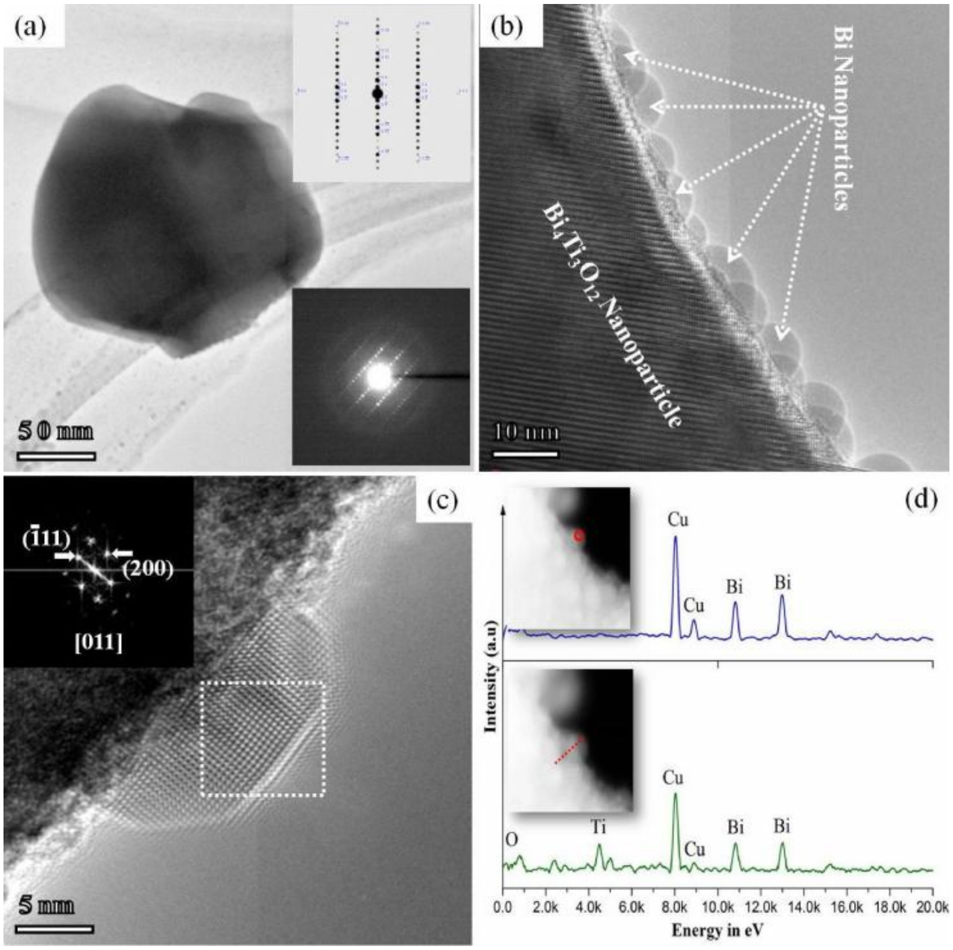


Figure S13. (a) Before e-beam irradiation of $Bi_4Ti_3O_{12}$ nanoparticle, the inset above and below are simulated and obtained SAED pattern, respectively. (b) TEM image of coexistence of Bi-$Bi_{4-x}Ti_3O_{12-y}$ nanocomposite material after e-beam irradiation. (c) HRTEM image of Bi metal nanoparticle and the inset is FFT pattern of the selected area (white rectangle). (d) EDS spectrum of the irradiated $Bi_4Ti_3O_{12}$.

## 9. Characterization techniques

The High Resolution Transmission Electron Microscopy (HRTEM), X-ray Energy Dispersive Spectroscopy (EDS) analysis and electron irradiations were performed in FEI Tecnai $G^2$ F30 microscope in which an electron beam produced from the field emission gun, operated at 300 KeV, current density (J) $2.02\times10^7$ A/$m^2$ (Electron beam parameters are given in the Table S1 and Fig. S9). The structural dynamic and electron irradiation effect events were recorded on hypercam Gatan CCD camera system with a rate of six frames per second at ambient temperature. The crystalline phases and structures of the materials were characterized with X-ray diffraction (XRD) using a Rigaku D/max 2200V/PC diffractometer with Cu Ka radiation ($\lambda$ = 1.54178 Å). X-ray photoelectron spectroscopy (XPS) using a Thermo Electron Corporation ESCA Lab250 spectrometer at 15kV and 150 W was employed to study the surface characteristic of irradiated and non-irradiated samples.

## 10. HRTEM image simulation

HRTEM simulation was carried out using the multislice method using the QSTEM program [2]. The simulated image of Pb particle was calculated from the model which was built on the basis of experimental image. The thickness and defocus values were chosen as 30nm and -34nm, respectively. The sample inclination was not taken into account in simulation.

## References:

1. W.H. Qi, *Physica B*, 2005, 368, 46-50.
2. C. Koch, Determination of Core Structure Periodicity and Point Defect Density along Dislocations. PhD Thesis (Arizona State University, 2002).
